# Development and Fibronectin Signaling Requirements of the Zebrafish Interrenal Vessel

**DOI:** 10.1371/journal.pone.0043040

**Published:** 2012-08-21

**Authors:** Chih-Hao Chiu, Chih-Wei Chou, Shinji Takada, Yi-Wen Liu

**Affiliations:** 1 Department of Life Science, Tunghai University, Taichung, Taiwan R.O.C; 2 Okazaki Institute for Integrative Bioscience, National Institutes of Natural Sciences, Okazaki, Aichi, Japan; 3 Department of Basic Biology, Graduate University for Advanced Studies (SOKENDAI), Okazaki, Aichi, Japan; Institute of Cellular and Organismic Biology, Taiwan

## Abstract

**Background:**

The early morphogenetic steps of zebrafish interrenal tissue, the teleostean counterpart of the mammalian adrenal gland, are modulated by the peri-interrenal angioblasts and blood vessels. While an organized distribution of intra-adrenal vessels and extracellular matrix is essential for the fetal adrenal cortex remodeling, whether and how an intra-interrenal buildup of vasculature and extracellular matrix forms and functions during interrenal organogenesis in teleosts remains unclear.

**Methodology and Principal Findings:**

We characterized the process of interrenal gland vascularization by identifying the interrenal vessel (IRV); which develops from the axial artery through angiogenesis and is associated with highly enriched Fibronectin (Fn) accumulation at its microenvironment. The loss of Fn1 by either antisense morpholino (MO) knockdown or genetic mutation inhibited endothelial invasion and migration of the steroidogenic tissue. The accumulation of peri-IRV Fn requires Integrin α5 (Itga5), with its knockdown leading to interrenal and IRV morphologies phenocopying those in the *fn1* morphant and mutant. *fn1b*, another known *fn* gene in zebrafish, is however not involved in the IRV formation. The distribution pattern of peri-IRV Fn could be modulated by the blood flow, while a lack of which altered angiogenic direction of the IRV as well as its ability to integrate with the steroidogenic tissue. The administration of Fn antagonist through microangiography exerted reducing effects on both interrenal vessel angiogenesis and steroidogenic cell migration.

**Conclusions and Significance:**

This work is the first to identify the zebrafish IRV and to characterize how its integration into the developing interrenal gland requires the Fn-enriched microenvironment, which leads to the possibility of using the IRV formation as a platform for exploring organ-specific angiogenesis. In the context of other developmental endocrinology studies, our results indicate a highly dynamic interrenal-vessel interaction immediately before the onset of stress response in the zebrafish embryo.

## Introduction

The adrenal gland is comprised of the outer three-layered cortex which synthesizes steroid hormones, and the inner medulla which produces catecholamines. Remodeling of the fetal adrenal cortex is featured by the differentiation and centripetal migration of cells from the highly-proliferative outer definitive zone (DZ) through the inner fetal zone (FZ) which is to regress and be replaced by the adult cortex [1]. The vascular architecture of the human fetal adrenal gland shows a clear centripetal pattern [2], indicating that the cortex remodeling and vascularization might be established in a coordinated fashion. The proliferation, differentiation, hormone responsiveness and apoptosis of the fetal adrenal cortex require an organized distribution of extracellular matrix (ECM) proteins including Laminin, Collagen IV and Fn [3], [4]. While Laminin is specifically expressed at the highly proliferative outer DZ, Collagen IV appears evenly distributed within the fetal adrenal gland, and Fn accumulates in a spatial gradient correlated with the direction of centripetal migration. It however remains unclear whether the Fn gradient functionally relate to the centripetal migration of adrenocortical cells, and whether it contributes to the vascular microenvironment along the FZ.

In the zebrafish, functional assembly of the interrenal gland, the teleostean counterpart of the mammalian adrenal cortex, is preceded by morphogenetic movements of the emerging interrenal tissue which are promoted by the endothelium [5], [6]. Bilateral primordial interrenal tissues marked by the expression of *ff1b* gene are specified within and segregated from the bilateral pronephric primordia [7], and are guided by migrating angioblasts to fuse at the midline at about 22- to 24- somite stage [5], [6]. Subsequently, rightward lateralization of the fused interrenal tissue takes place in parallel with and is modulated by the formation of axial vessels, resulting in a single interrenal tissue juxtaposed in-between the dorsal aorta (DA) and right branch of the posterior cardinal vein (PCV) by 28 hours postfertilization (hpf), when the steroidogenic activity is initiated. The molecular nature of the endothelium-derived signal that promotes early interrenal morphogenetic movements has remained unknown. In addition, it was unclear whether the endothelial signaling also plays a role during the subsequent functional assembly of interrenal gland, which is marked by the integration of steroidogenic and chromaffin cells. Assembly of the interrenal gland is initiated at around 2 days postfertilization (dpf) and completed by 3 dpf, during which interrenal cells disperse and migrate from right side of the DA toward the midline, and situated immediately caudal to the vascularized pronephric glomerulus, where they intermingle with chromaffin cells derived from the trunk neural crest [8], [9]. In this study, we discovered and characterized the interrenal vessel (IRV) that invaginated into the interrenal tissue, with its development temporally correlated with central migration of steroidogenic interrenal cells during the period of interrenal gland assembly. Interestingly, Fn is highly enriched around the sprouting site and tip cell of the IRV, as well as at the interface between the IRV and migrating interrenal cells, implying a possible role of Fn in coordinating processes of the IRV angiogenesis and the interrenal migration.

The reciprocal interaction between endothelial cells (EC) and their surrounding microenvironment has been described for the development and function of a diversity of organ systems including heart, endodermal, neural and hematopoietic tissues, and is implicated in pathophysiology such as tumorigenesis [10], [11]. A common feature of the vascular microenvironment has been hypothesized to be the basement membrane that contains Laminins, Collagen IV and Heparan Sulphate Proteoglycans, which might act in concert with proangiogenic factors to orchestrate processes such as proliferation and secretion of pancreatic β-cells which lack a basement membrane structure [12], [10]. Fibronectin (Fn), a major adhesive molecule of the ECM, is often associated with the vascular basement membrane in the cardiovascular organogenesis [13]. The genetic study in mice has also revealed a role of Fn for mediating the interaction between endothelial and peri-endothelial cells in the heart and the DA [14]. Fn is involved in a wide spectrum of cellular processes, through its interaction with the Integrin receptor family, to mediate cell-substratum adhesion and spreading, cell migration, and cytoskeletal organization etc [15], [16]. In chick embryos, Fn accumulates at the interface between the endoderm and mesoderm and is implicated in myocardial migration [17]. Likewise, in zebrafish, Fn deposition is seen around the myocardial precursors, as well as the midline region between the endoderm and endocardial precursors [18]. Consistent with the phenotype of Fn null mouse embryo [14], the zebrafish *fn* mutant (*natter*) demonstrates normal myocardial specification and yet defective myocardial migration [18], with the effect of Fn on myocardial fusion negatively regulated by Hand2 [19]. In the zebrafish embryo, Fn also accumulates among the aggregating angioblasts and around the vascular endothelium [20], suggesting an early role of Fn in the vascular tube lumen formation. However, the role of Fn in the zebrafish vascular development has not been well described. *fn* mRNA expression is deficient in the endothelium-free *cloche* (*clo*) mutant [18], [21], implying that the Fn deposition around the developing vasculature might be generated either by the endothelium, or through an interaction between endothelial and peri-vascular cells. Our results showed an enrichment of Fn around the sprouting IRV which might be originated from the endothelium. While the Fn deficiency in the early embryo did not significantly affect general vascular assembly and major angiogenic processes including those of intersegmental vessel (ISV) and kidney glomerulus, the angiogenic invasion of IRV into the interrenal tissue was repressed. In parallel, the morphogenetic movement of the interrenal tissue was defective in the *fn* morphant or mutant. The inhibition of Integrin α5 (Itga5), the putative receptor of Fn, led to defects of both interrenal migration and IRV formation, phenocopying those in the Fn-deficient embryo. We have also shown that the effect of disrupted Fn-Itga5 signaling on the IRV formation was not due to general cardiovascular defects associated with perturbed blood flow. Interestingly, a lack of blood flow did not affect growth of the IRV; which however led to altered Fn association and angiogenic directionality of the IRV, as well as disrupted migration of steroidogenic cells. The blood flow-independent effect of Fn signaling on the interrenal morphogenesis was supported by the administration of Fn antagonist through microangiography, which was able to reduce both IRV angiogenesis and interrenal migration partially. Our study thus demonstrated how the Fn-enriched microenvironment modulates the growth of IRV whose angiogenic pattern is flow-sensitive. The IRV and its associated Fn might in turn promote the medial extension of steroidogenic tissue during interrenal organ development.

## Methods

### Ethics Statement

All of the zebrafish-use protocols in this research were reviewed and approved by the Institutional Animal Care and Use Committee of Tunghai University (IRB Approval NO. 96-05).

### Zebrafish Husbandry

Zebrafish (*Danio rerio*) were raised under standard conditions [22] in accordance with IACUC-approved protocols. Embryos were obtained from natural crosses of wild-type, transgenic or heterozygous mutant fish, and staged as previously described [23]. The following lines were used in this study: *fn1^kt259^* [24], *Tg(kdrl:EGFP)^s843^* (gift of Didier Stainier, University of California, San Francisco, USA), and *Tg(ff1bEx2:GFP)* [6] (gift of Dr. Woon-Khiong Chan, NUS, Singapore).

### 3β-Hydroxysteroid Dehydrogenase (3β-Hsd) Staining, *in situ* Hybridization (ISH) and Immunohistochemistry (IHC)

Embryos to be subject to histological analysis were treated with 0.03% phenylthiourea (Sigma) from 12 hpf onwards to inhibit pigment formation. 3β-Hsd activity staining, ISH and IHC were performed essentially according to [25], [5] and [18] respectively with modifications.

To delineate the morphology of steroidogenic interrenal tissue, histochemical staining for 3β-Hsd enzymatic activity was performed on whole embryos, and Nomaski images were captured using an Olympus BX51 microscope system. For simultaneous analysis of interrenal steroidogenic activity and endothelial GFP fluorescence on a whole-mount or sectioned embryo, 3β-Hsd activity signals were captured using transmitted light, while the fluorescent signals captured as either single confocal sections or the projection of a Z-stack, with Argon 488-nm laser connected to a Zeiss Axioplan II microscope equipped with LSM510 (Carl Zeiss). Image processing and analysis, including length quantification of the IRV, was performed using the LSM 510 version 3.5 software.

For whole-mount ISH assays, digoxigenin-labeled antisense riboprobes were synthesized from linearized plasmids of *wt1a*, *wt1b*, *dβh*, *fn1*, *fn1b* and *itga5* respectively. Fluorescein-labeled antisense riboprobes were synthesized from linearized *ff1b* plasmids. DIG-labeled riboprobes were detected with alkaline phosphatase conjugated anti-DIG antibody (Roche) while fluorescein-labeled probes were detected by alkaline phosphatase conjugated anti-fluorescein antibody (Roche). Visualization was performed either with BCIP/TNBT (Millipore), or with Fast Red (Roche). For two-color ISH, inactivation of the first antibody was performed by heating the stained embryos at 65°C for 30 min. Stained embryos were post-fixed in 4% paraformaldehyde (PFA) in PBS and washed in PBST (PBS supplemented with 0.1% of Tween 20). The stained embryos were subject to further processing with a Leica VT1000M vibratome into 50 µM sections, or to yolk-sac removal and flat-mount analysis. The specimen were cleared in 50% glycerol in PBS, mounted on glass slides and photographed under Nomaski optics on a Zeiss Axioplan II microscope equipped with LSM510.

For IHC experiments performed on 3β-Hsd activity-stained *Tg(kdrl:GFP)^s843^* embryos, or on *Tg(ff1bEx2:GFP)* embryos, fixed embryos were embedded in 4% NuSieve GTG low-melting agarose, cut into 100 µM sections with a Leica VT1000M vibratome, and permeabilized with PBS containing 1% Triton X-100, before antibody staining. Anti-Fn staining was performed using the rabbit polyclonal anti-Fn (Sigma) at 1∶200 in PBS containing 1% Triton X-100 and 10% Fetal Calf Serum. Anti-phosphorylated Focal Adhesion Kinase (pFAK) staining was performed using the mouse anti-human FAK (pY397) (BD Transduction Laboratories) at 1∶100 in PBDT (1% BSA, 1% DMSO, 0.1% Triton X-100 in PBS). Dylight^TM^594-conjugated goat anti-rabbit or anti-mouse IgG was used as the secondary antibody at a 1∶200 dilution. Images of vibratome-sectioned embryos were captured with the confocal microscopy.

### Microinjection of Antisense Morpholino Oligonucleotides (MOs)

MOs were synthesized at Genetools LLC, diluted in 1×Danieau solution, and injected into one- to two-cell stage embryos by using a Nanoject (Drummond). *fn1*MO (5′ – CAC AGG TGC GAT TGA ACA CGC TAA A - 3′) [24] was injected at the dosage of 1.2 pmole per embryo. To target *fn1b* expression, 0.6 pmole each of *fn1b*MO1 (5′ – TAC TGA CTC ACG GGT CAT TTT CAC C –3′) and *fn1b*MO2 (5′ – GCT TCT GGC TTT GAC TGT ATT TCG G - 3′) [26] were co-injected into each embryo. To target *itga5* expression, 0.3 pmole each of *itga5*MO1 (5′ – CAT AGT AAC CGA TGT ATC AAA ATC C - 3′) and *itga5*MO2 (5′ – ACT GCT TTA TTA AAC TTC TTT CAC A - 3′) [24] were co-injected into each embryo. *tnnt2*MO (5′-CAT GTT TGC TCT GAT CTG ACA CGC A-3′) [27] was injected at the dosage of 1.0 pmole per embryo.

### Microangiography

Microangiography for delineating the blood flow pattern or for RGD heptapeptide injections was performed essentially according to [28] with modifications. For capturing the blood flow pattern at the interrenal region, Rhodamine-conjugated dextran (Tetramethylrhodamine, 2.000.000 MW, Invitrogen) was injected into the PCV of *Tg(ff1bEx2:GFP)* embryos slightly anaesthetized with tricaine. The injected embryos were fixed and mounted in 3% methyl cellulose, and the fluorescent signals of *ff1b* promoter-driven GFP and dextran were captured using the confocal microscopy.

In the drug treatment experiments, either RGD heptapeptide (Sigma #G1269, reconstituted to 10 mM in filter-sterilized embryo medium) or BSA control was injected into the PCV of *Tg(kdrl:GFP)^s843^* embryos at 1.5 dpf. Injected embryos were cultured in the embryo medium, and harvested at 52 hpf for whole-mount 3β-Hsd activity staining. The stained embryos were subject to vibratome sectioning and confocal microscopy analysis.

### Statistical Analysis

All quantitative data are expressed as the mean±SE of the mean (SEM). Statistical analysis of the data was performed using analysis of variance, followed by Student’s t-test. A probability of *P*<0.05 was considered statistically significant.

## Results

### The Peri-interrenal Endothelium Invades the Steroidogenic Interrenal Tissue by Angiogenic Sprouting

**Figure 1:**
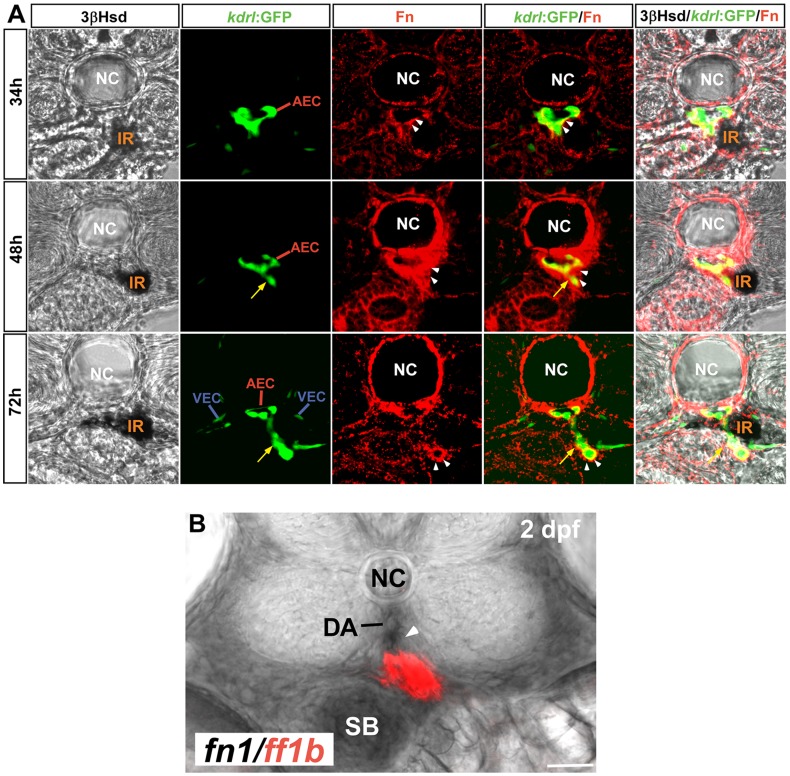
The formation of IRV by angiogenic sprouting from the DA. (A) Transverse sections of *Tg(kdrl:GFP)^s843^* embryos at 34, 48 and 72 hpf visualized for 3β-Hsd activity (black), GFP (green) and Fibronectin (red). All sections are oriented with the posterior end toward top of page. While the IRV sprouting from the DA (yellow arrows) invades the interrenal tissue, steroidogenic interrenal cells form a protruding extension which migrates toward and across the central midline. Fibronectin (white arrowheads) accumulates at the interface between the endothelium and the interrenal tissue, and around the tip of growing IRV endothelium. (B) *fn1* transcripts (black; indicated by white arrowhead) were detected around and ventral to the DA on the transverse section at the level of *ff1b*-expressing interrenal tissue (red) in a 2 dpf embryo. Abbreviations: notochord (NC), interrenal tissue (IR), arterial endothelial cell (AEC), venous endothelial cell (VEC), dorsal aorta (DA), swim bladder (SB). Scale bar is 50 µM.

Along with the sprouting of IRV from the DA, interrenal cells on the right side of the midline migrated along the ventral side of the DA, and formed intimate interactions with the DA as well as the angiogenic IRV (Fig. 1). The temporal coincidence between the IRV angiogenesis and the central migration of interrenal cells implied that the IRV and/or its derived signal could directly or indirectly induce the migration of interrenal cells. Consistent with the findings of Jin et al [20], Fn was found to deposit around the DA. Interestingly, Fn was highly enriched at the peri-vascular region where the IRV was budded off from the ventral side of the DA, and along the tip of IRV extension. Temporally, the peri-endothelial Fn deposition in the interrenal region demonstrated an increase from 34 to 48 hpf, yet subsequently a decrease by 72 hpf. This apparently transient Fn expression might be caused majorly by the dynamic Fn accumulation throughout the process of IRV sprouting. The increasing Fn deposition from 34 to 48 hpf was temporally and spatially correlated with the initiation of IRV sprouting from the DA. Although a rich Fn accumulation was no longer found near the IRV sprouting site by 72 hpf, it could instead be detected around the tip of the extending IRV. This suggested that endothelial cells at the front of actively sprouting IRV synthesize Fn which in turn supports the angiogenic activity. Also, the peri-vascular Fn deposition might serve to ensure a tight association between the EC and the interrenal tissue during the early stage of IRV angiogenesis.

Topographically, the IRV was close to yet distinct from the more anterior vessels which were also derived from the ventral DA, including the kidney glomerulus and the anterior mesenteric artery (AMA) (see Fig. S1A). After spanning through the interrenal tissue, the IRV was connected to the posterior end segment of the AMA where two branches of swim bladder arteries branched out (see Fig. S1A, B and Video S1). The posterior end segment of the AMA is also connected to the supraintestinal artery which further links to the subintestinal vessel (SIV) [28]. The blood flow through the IRV was established by 3 dpf (see Fig. S1C), implying that the steroids synthesized from the embryonic interrenal tissue could be transported through the IRV and supply to the developing swim bladder and intestinal system.

While the Fn expression was enriched at the interface between vascular and migrating interrenal cells during invagination of the IRV, it is possible that the endothelium of the DA and/or the IRV could modulate morphogenesis of the interrenal tissue through synthesizing Fn. It is therefore essential to verify the origin of *fn1* synthesis at the peri-interrenal area. Double ISH analysis displayed that the *fn1* transcript at the peri-interrenal region was enriched at the DA and the IRV, indicating its synthesis from the developing vessels (Fig. 1B, arrowhead). As the mural cell population accumulates at the ventral side of the DA only from 72 hpf onwards [29], the peri-interrenal *fn1* RNA and protein earlier than 3 dpf could neither be synthesized by vascular mural cells, nor be resulted from an interaction between endothelial and mural cells. It thus suggested that the DA- and IRV-associated Fn deposition in the embryo was likely originated from the endothelium.

### The Absence of Fn was Neither Inhibitory to Angiogenesis of the Intersegmental Vessel (ISV) and the Kidney Glomerulus, nor to the Association between Interrenal and Endothelial Cells

**Figure 2:**
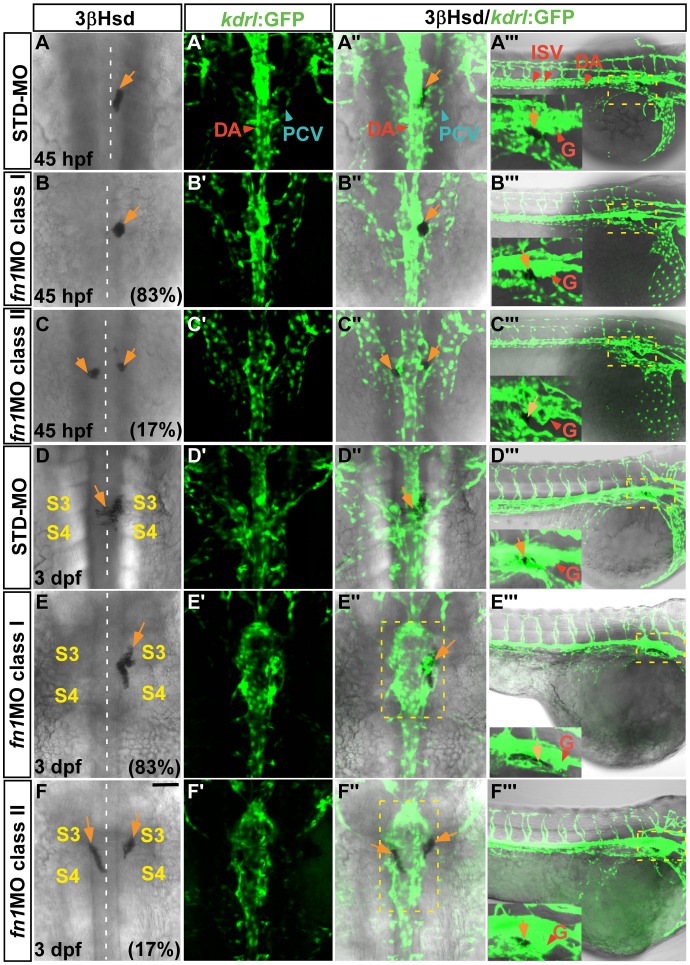
The vascular phenotype and interrenal-endothelial interaction in the *fn1* morphant. Sets of confocal images display the interrenal tissue as detected by 3β-Hsd activity staining (orange arrows), and the midtrunk vasculature by green fluorescence, of 45 hpf and 3 dpf *Tg(kdrl:EGFP)^s843^* embryos injected with either STD-MO or *fn1*MO. Panels (A–F, A’–F’, A’’–F’’) are dorsal views showing the midtrunk of the representative embryo for each phenotypic class, with anterior oriented to the top; while panels (A’’’–F’’’) lateral views of the same embryo with anterior to the right. The outlined peri-interrenal areas in E’’ and F’’ indicate enlarged DA segments. The outlined glomerular (G) and interrenal regions in (A’’’–F’’’) are magnified and annotated in the insets. The angiogenesis of ISV and kidney glomerulus, as well as the association between interrenal and endothelial cells, are not inhibited upon the absence of Fn. Abbreviations: dorsal aorta (DA), posterior cardinal vein (PCV), intersegmental vessel (ISV), glomerulus (G), the third somite (S3), the fourth somite (S4). Scale bar is 50 µM.

**Figure 3:**
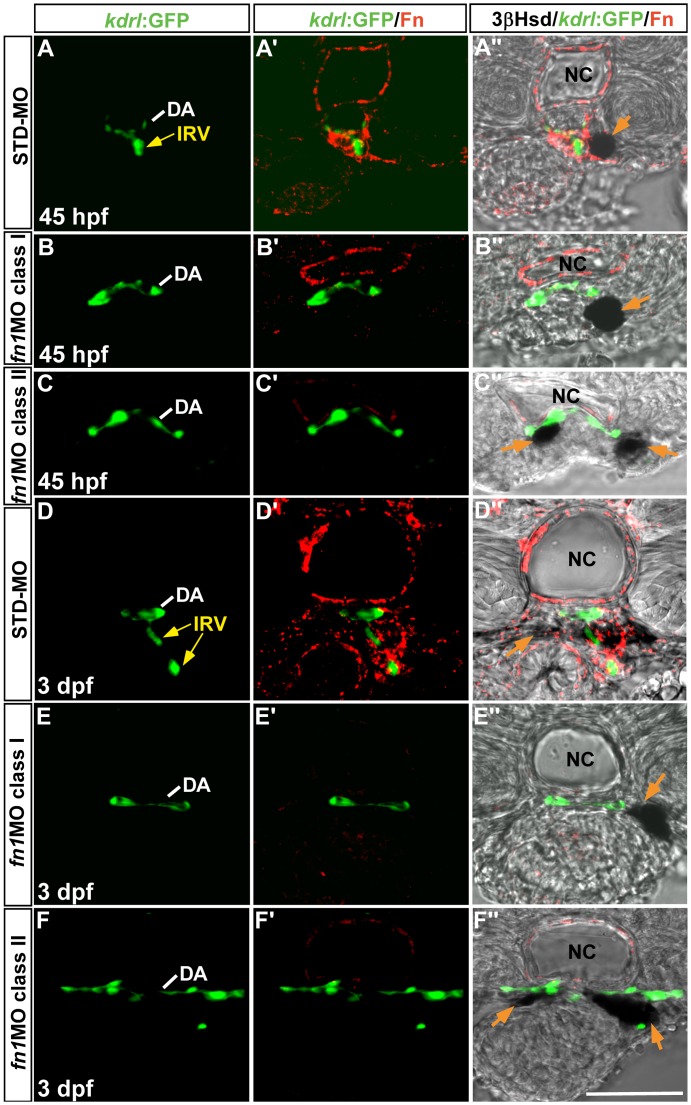
The effect of *fn1* knockdown on the interrenal tissue and the peri-interrenal vasculature. Transverse sections of *Tg(kdrl:GFP)^s843^* embryos injected with either STD-MO (A–A’’, D–D’’) or *fn1*MO (B–B’’, C–C’’, E–E’’, F–F’’) were assayed for 3β-Hsd activity (black), GFP (green) and Fn expression (red). All sections are oriented with the posterior end toward top of page. The IRV structure of a 3 dpf control embryo (D’–D’’) appeared to be discontinuous on a single confocal section, because the IRV at this stage extended slightly anteriorward and then posteriorward, after spanning ventrally through the interrenal tissue and before connecting to the AMA segment (Video S1). The formation of IRV is defective in the absence of peri-vascular Fn. Yellow arrows indicate the IRV sprouting from the DA. Orange arrow denotes the interrenal tissue. Abbreviations: dorsal aorta (DA), interrenal vessel (IRV), notochord (NC). Scale bar is 50 µM.

The rich deposition of Fn at the peri-IRV region prompted us to explore whether Fn is involved in morphogenesis of the IRV. For this purpose, the morpholino oligo against *fn1* was injected into the *Tg(kdrl:GFP)^s843^* embryo, where the EGFP expression driven by the promoter of *kinase insert domain receptor like* gene marks the endothelium [20], for analyzing both interrenal and vascular phenotypes (Fig. 2). The *fn1* morphant fully phenocopied the *fn1* mutant by showing the hindbrain natter and disrupted somite boundary phenotypes [18], [24]. At both 45 hpf and 3 dpf, 83% of the *fn1* morphants demonstrated an interrenal phenotype where no medial extension of steroidogenic tissue cluster was detected (class I) (n = 47 and 30 for 45 hpf and 3 dpf respectively), while the rest of the morphants showed the class II phenotype. Meanwhile, STD-MO injected controls displayed the migratory interrenal phenotype at 45 hpf (n = 16/19) and 3 dpf (n = 16/16) respectively. At 45 hpf, the general morphology of axial vasculature appeared not significantly affected in *fn1* morphants, in either class I or class II phenotype (Fig. 2A’–A’’’,B’–B’’’, C’–C’’’). At 3 dpf, the dorsal view of the *fn1* morphant displayed enlargement of the DA in proximity to the interrenal region, at the midtrunk level from 3rd to 4th somite (Fig. 2 E”, F”). The enlargement of the DA could be due to a flattened and dilated lumen shape (Fig. 3 B’, C’, E’, F’).

For both classes of *fn1* morphants, angiogenic processes of either ISV or kidney glomerulus were not inhibited (Fig. 2B’’’, C’’’), although the first four pairs of ISV in the *fn1* morphant displayed an irregular sprouting pattern (Fig. S2) possibly correlated with a disrupted formation of anterior somites upon the absence of Fn1 [24]. In addition, the loss of Fn did not lead to an entire repression of SIV formation, whose angiogenic pattern was nevertheless abnormal and could not form a barrel-shaped vascular structure typical for the SIV at 3 dpf. Fn accumulates between the lateral plate mesoderm and the gut [30], therefore it is possible that Fn might participate in the tissue microenvironment supporting the SIV development. It however remains unclear whether the defective SIV structure in the *fn1* morphant was due to a disrupted gut morphogenesis, or to a loss of the Fn microenvironment.

Interestingly, the tight association between steroidogenic and endothelial cells appeared unaffected in the *fn1* morphant from whole-mount views. Even at 3 dpf when the vascular morphology was perturbed at the peri-interrenal area, interrenal tissues showing either class I or class II phenotype remained associated with the axial vasculature. The interaction between interrenal tissue and its adjacent endothelium has been found to initiate before the assembly of axial vessels [5], and the interaction persists even upon the disruption of either axial artery or vein [6]. Nevertheless, defective assembly of the axial vasculature causes defects in either bilateral fusion or lateral relocalization of interrenal tissues. It is therefore possible that, in *fn1* morphants, a defective vascular structure contributes to the aberrant interrenal morphology. Hence we performed vibratome sectioning and analyzed how the loss of Fn would affect the pattern of peri-interrenal vessels.

### Angiogenic Growth of the IRV into the Steroidogenic Interrenal Tissue was Repressed in the Absence of Fn

**Figure 4:**
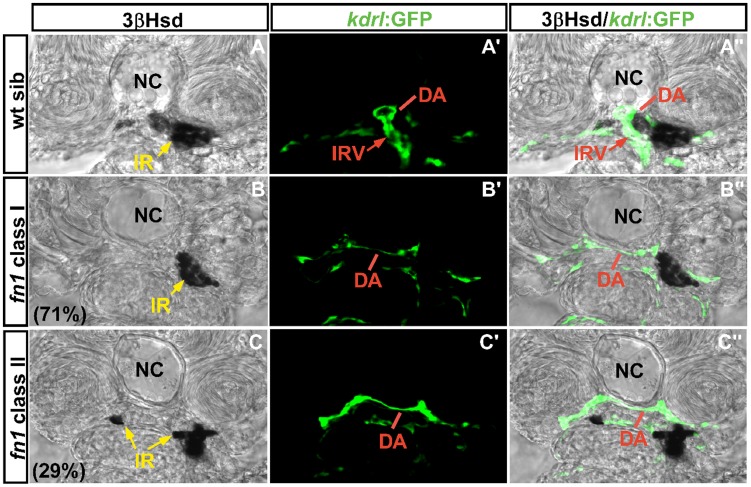
The interrenal tissue and peri-interrenal vasculature phenotypes in the *fn1* mutant. Transverse sections of *fn^kt259^;Tg(kdrl:EGFP)^s843^* embryos and their wild-type siblings at 3 dpf were visualized for 3β-Hsd activity (black) and GFP (green). All sections are oriented with the posterior end toward top of page. The images for wild-type siblings (A–A’’) were acquired by focusing on the sprouting point of the IRV from the DA, and hence its surrounding steroidogenic cells were masked by the endothelial structure on panel A. The IRV angiogenesis and interrenal morphogenetic movements are severely disrupted in the *fn* mutant. Yellow arrow, interrenal tissue (IR). Abbreviations: notochord (NC), interrenal tissue (IR), dorsal aorta (DA), interrenal vessel (IRV).

While the peri-vascular Fn in the control embryo was enriched around the budding site of IRV and along the path of interrenal migration, no Fn deposition was found around the equivalent area in *fn1* morphants (Fig. 3). Consistent with the whole-mount analysis in Fg. 2, the interrenal tissue in *fn1* morphants displaying either class I or II phenotypes stayed associated with the DA. However, no angiogenic growth of the IRV was detected in *fn* morphants at either 45 hpf or 3 dpf (Fig. 3B”, C”, E”, F”), despite a normal angiogenic pattern for the ISV and the kidney glomerulus (Fig. 2B’’’, C’’’, E’’’, F’’’). At 45 hpf, examination from transverse sections revealed that the IRV was absent in both class I and II *fn1* morphants (Fig. 3B–B”, n = 15/19 for class I; Fig. 3C–C”, n = 7/7 for class II), while the IRV formation was normal in all STD-MO injected controls (Fig. 3A–A”, n = 19). Likewise, both class I and II *fn1* morphants at 3 dpf displayed no IRV formation (Fig. 3E–E”, n = 10/11 for class I; Fig. 3F–F”, n = 9/11 for class II), while all STD-MO injected controls showed normal IRV formation (Fig. 3D–D”, n = 16). Summarized from the results of Fig. 2 and 3, we concluded that the loss of endothelium-associated Fn did not appear to affect endothelial association of the interrenal tissue; it however impaired growth and penetration of the IRV into the interrenal gland during its functional assembly. As the loss of IRV was detected as early as 45 hpf, it could be due to a failure for the DA to initiate IRV sprouting, rather than being the result of vessel regression. The interrenal migration and IRV formation phenotypes in the *fn1* morphant phenocopied those in the *fn1* mutant which was outcrossed to *Tg(kdrl:GFP)^s843^* for marking the peri-interrenal vasculature (Fig. 4). The angiogenesis of IRV was severely disrupted in all of the class I (B–B”) and II (C–C”) *fn1* mutants (71% and 29% of *fn1^−/−^* respectively; no of *fn1^−/−^ = *31).

**Figure 5:**
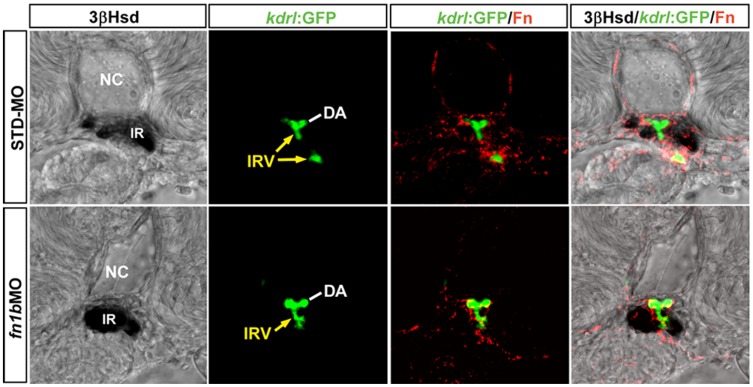
The effect of *fn1b* knockdown on the interrenal tissue and the peri-interrenal vasculature. Transverse sections of *Tg(kdrl:EGFP)^s843^* embryos were injected with either STD-MO or *fn1b* antisense morpholino, harvested at 3 dpf and visualized for 3β-Hsd activity (black), GFP (green) and Fn expression (red). All sections are oriented with the posterior end toward top of page. The IRV structure (yellow arrows) of a 3 dpf control embryo appeared discontinuous on a single confocal section because the IRV at this stage, after spanning through the interrenal tissue (IR), curved anteriorward and then posteriorward before connecting to the AMA segment. Fn doposition in the interrenal region, as well as the interrenal migration and angiogenesis, remain unperturbed in the *fn1b* morphant. The Fn expression and IRV morphology in the *fn1b* morphant shown here is a representative of 10 samples exhibiting a disruption of somite boundaries characteristic of the Fn1b-deficient embryo. Abbreviations: notochord (NC), interrenal tissue (IR), dorsal aorta (DA), interrenal vessel (IRV).

In addition, *fn1b*, another *fn* gene that cooperates with *fn1* to mediate somite boundary formation in the embryo, did not contribute to the Fn deposition at the peri-interrenal area (Fig. 5). The knockdown of *fn1b* caused a disruption of somite boundary formation as reported previously [31], yet did not lead to significant loss of Fn at the peri-IRV region. Moreover, the interrenal tissue in 88% of *fn1b* morphants (n = 64) demonstrated a normal central migration phenotype, while the IRV formation was not repressed. Consistently, the mRNA of *fn1b* was not detectable at either the DA or the IRV (Fig. S3), supporting that *fn1* could be the only *fn* gene that participates in the regulation of IRV formation and interrenal cell migration.

### The *itga5* Morphant Displayed Reduced Fn Accumulation and Interrenal Morphologies that Phenocopied the *fn1* Morphant

**Figure 6:**
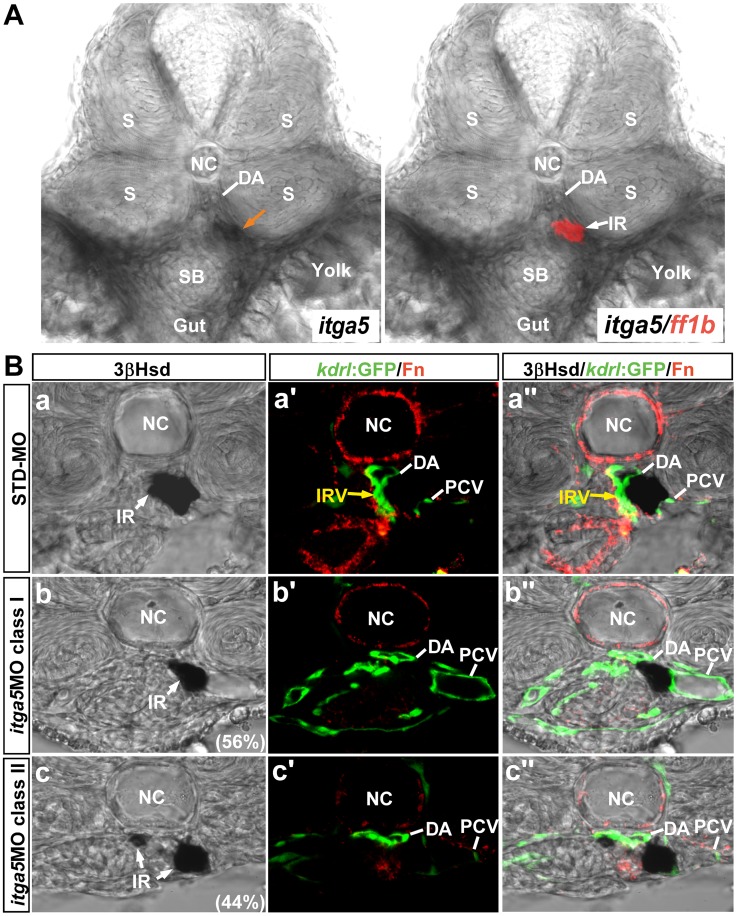
The expression and phenotype of *itga5* at the level of interrenal tissue at 2 dpf. (A) The double ISH assay revealed that *itga5* was expressed in the interrenal area and at the non-somitic mesodermal cells around the DA, the swim bladder (SB) and the gut. (B) The effect of *itga5* knockdown on the interrenal tissue (IR, white arrows) and the peri-interrenal vasculature. Transverse sections of *Tg(kdrl:GFP)^s843^* embryos injected with either STD-MO (a–a’’) or *itga5* MOs (b–b’’, c–c’’) and assayed for 3β-Hsd activity (black), GFP (green) and Fn expression (red). All sections are oriented with the posterior end toward top of page. The accumulation of peri-vascular Fn and the formation of IRV (yellow arrows) are both disrupted upon the knockdown of *itga5* expression. Abbreviations: notochord (NC), somite (S), dorsal aorta (DA), swim bladder (SB), interrenal tissue (IR), interrenal vessel (IRV), posterior cardinal vein (PCV).

Itga5 has been identified to be crucial for the initiation of fibronectin fibrillogenesis, which was essential for maintaining somite boundaries in the zebrafish embryo [24]. To verify whether Itga5 also participates in the IRV formation and interrenal migration, we further checked the expression and phenotype of *itga5* in the interrenal region. The transcripts of *itga5* at the midtrunk was distributed at the non-somitic mesodermal cells that surround the DA, the swim bladder and the gut (Fig. 6A), which appeared complementary to those of *fn1* enriched at the DA, the IRV and the swim bladder (Fig. 1B). It is noteworthy that *itga5* was also expressed at the interrenal area, implicating the possibility for Fn-Itga5 signaling to function at the boundary between vascular and interrenal cells. All the *itga5* morphants examined showed a defective somite boundary formation during early somitogenesis (n = 27) and phenocopied the reported *itga5* mutant [32], [24]. Consistent with Koshida et all [24] that the Fn accumulation at the somitic boundary requires Itga5, the *itga5* morphant at 2 dpf displayed a significant loss of Fn at the midtrunk (Fig. 6Bb’,Bc’). Albeit to a lesser extent than that in the *fn1* morphant and mutant, the DA structure at the level of interrenal tissue area appeared dilated and flattened in the *itga5* morphant, plausibly due to a perturbed cardiac flow. In agreement with the loss of Fn deposition at the level of interrenal tissue in the *itga5* morphant, the *itga5* morphant phenocopied the *fn1* morphant and mutant in terms of the IRV formation and the interrenal migration. The *itga5* morphants displayed either defective central migration (class I; n = 15/27) or unsuccessful bilateral fusion (class II; n = 12/27) (Fig. 6B), and a hindered growth of the IRV was found in both classes of *itga5* morphants (n = 11/15 for class I and 9/12 for class II), while the STD-MO injected controls demonstrated normal interrenal migration and IRV formation (n = 8).

**Figure 7:**
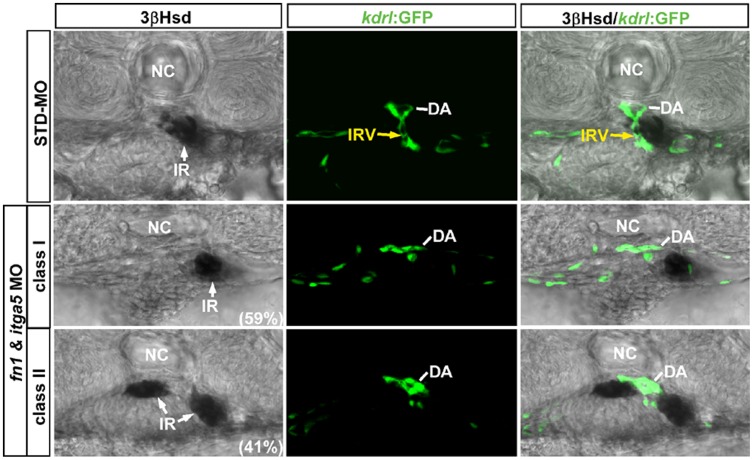
The effect of *fn1* and *itga5* double knockdown on the interrenal tissue and the peri-interrenal vasculature. Transverse sections of *Tg(kdrl:EGFP)^s843^* embryos injected with either STD-MO or *fn1/itga5* antisense morpholinos, harvested at 2 dpf and visualized for 3β-Hsd activity (black) and GFP (green). All sections are oriented with the posterior end toward top of page. The *fn1/itga5* double morphant phenocopied either *fn1* or *itga5* morphant in both migration and vessel formation of the interrenal tissue (IR, white arrow). Yellow arrows indicate the IRV sprouting from the DA. Abbreviations: notochord (NC), interrenal tissue (IR), interrenal vessel (IRV), dorsal aorta (DA).

A simultaneous knockdown of Fn and Itga5 by coinjecting the morpholinos against each gene led to a similar two classes of interrenal migration phenotypes as in the *fn1* and *itga5* experiments (Fig. 7). However, the percentage of class II phenotype among the double morphants (59%, n = 58) was higher than that of the class I phenotype (41%), suggesting that the simultaneous repression of *fn1* and *itga5* expressions could lead to a more profound effect on the early fusion process of bilateral interrenal tissues. Meanwhile, 90% of randomly selected embryos from each interrenal morphology class of double morphants (n = 10 from each class) demonstrated no formation of the IRV, which again supported that Fn and its putative receptor Itga5 function cooperatively for the IRV sprouting from the DA. To further validate the genetic interaction between Fn1 and Itga5 for the interrenal development, we also checked the localization and activation of Integrin signaling component Focal Adhesion Kinase (FAK) in both *fn1* and *itga5* morphants (Fig. S4). The results showed that the phosphorylated and thus activated form of FAK, pFAK, was detected at both interrenal and peri-interrenal regions, which was severely disrupted in both *fn1* and *itga5* morphants. The severely-disrupted FAK activation in both *fn1* and *itga5* morphants strengthens the notion that Fn1 and Itga5 function cooperatively for the interrenal morphogenesis and vessel formation.

### Anigogenic Directionality but not Growth of the IRV, as well as its Interaction with the Interrenal Tissue, were Perturbed in the Absence of Blood Flow

**Figure 8:**
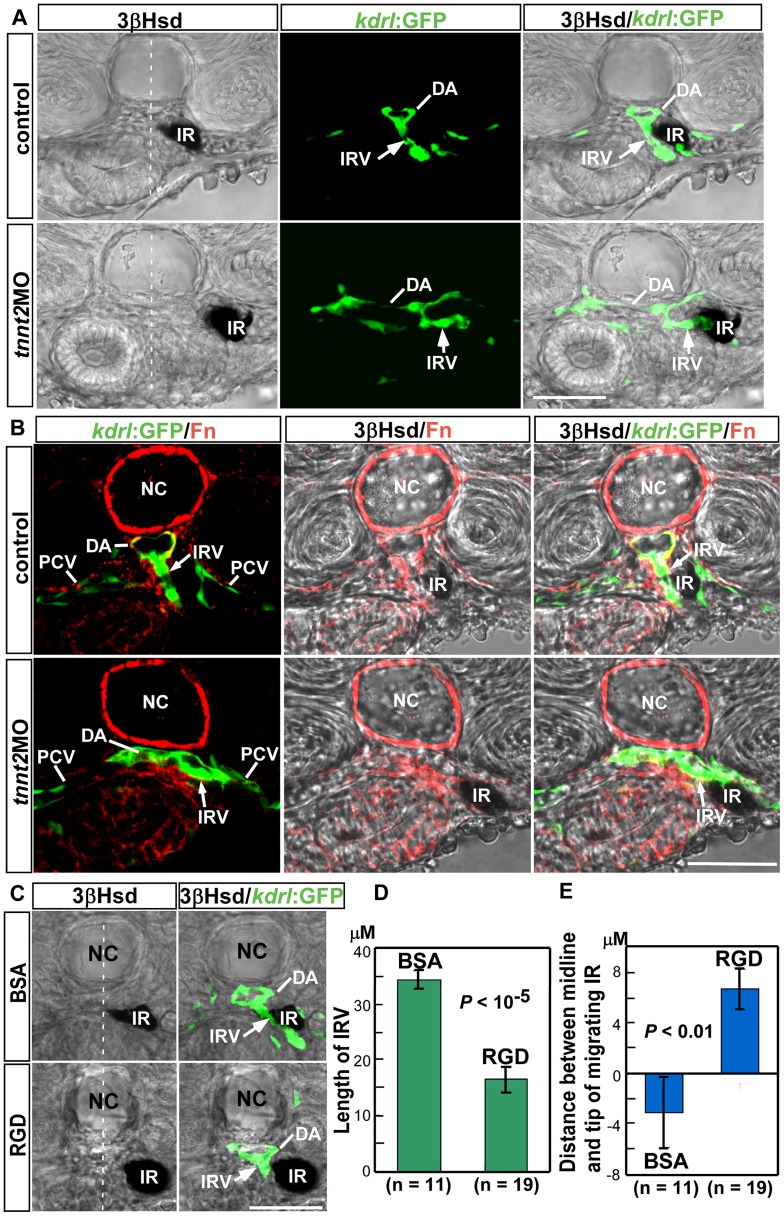
The effect of *tnnt2a*MO and RGD peptide treatment on the formation of IRV. (A) Blockage of blood flow by the *tnnt2a*MO injection into *Tg(kdrl:GFP)^s843^* embryos led to a collapsed and enlarged phenotype of the DA at 48 hpf, yet did not inhibit the formation of IRV (white arrows). (B) While Fn (red) was tightly aggregated around the IRV in the control embryo at 48 hpf, in the *tnnt2a* morphant it was loosely deposited around the IRV which extended toward the lateral rather than the ventral side. (C) RGD treatment via microangiography into *Tg(kdrl:GFP)^s843^* embryos at 1.5 dpf and harvested at 52 hpf showed that antagonizing the Fn function led to stunted IRV formation and retarded steroidogenic cell migration as compared with the BSA injected control embryo, which was confirmed by quantification of the length of IRV in (D) and of the distance between the midline and the tip of migrating interrenal tissue in (E). (D) The length of IRV was checked with confocal Z-stacks covering the full range of IRV growth, and measured from single focal planes displaying the maximal range of ventrally extending IRV. (E) The distance between the midline and the migrating tip of steroidogenic tissue is designated as positive if the tip of migrating cells has not reached the midline, and as negative if the tip has migrated across the midline. Abbreviations: interrenal tissue (IR), dorsal aorta (DA), interrenal vessel (IRV), notochord (NC), posterior cardinal vein (PCV). Scale bar is 50 µM.

The dilated DA lumen structure in the *fn1* morphant and mutant implied the possibility that the loss of IRV formation in the *fn1*-deficient embryo might be due to aberrant maturation of the DA. Fn is required for the integrity of the myocardial epithelia, and its absence resulted in cardiac bifida and a loss of blood flow [18]. Furthermore, recent studies have shown that the blood flow, through activating the nitric-oxide signaling, regulates maturity of the DA which is essential for establishing aorta-gonads-mesonephros vascular niche [33]. Therefore, we checked whether the loss of blood flow alone was sufficient to inhibit the IRV growth, by knocking down the *cardiac troponin T2a* (*tnnt2a*) gene which is essential for assembly of sarcomere and heart contractibility [27]. While all the *Tg(kdrl:GFP)^s843^* embryos injected with the morpholino oligo against *tnnt2a* displayed no blood flow (n = 20), transverse sections revealed a dilated DA structure highly resembling that in the *fn1* mutant and morphant (Fig. 8A; Fig. 3, 4). Interestingly, the formation of IRV was not inhibited in the *tnnt2a* morphant where the DA was hypomorphic, meaning that the loss of IRV in the *fn1* morphant or mutant was not entitled to secondary effects resulted from a lack of circulation and its resulting vascular defects. Nevertheless, as compared with the IRV in the control embryo which grew toward the ventral direction, the IRV in the *tnnt2a* morphant was directed to a more lateral position to reach the interrenal tissue. Also, the interrenal tissue did not interact intimately with the sprouting site of IRV, and was displaced more laterally to the midline. While both *fn1* and *tnnt2a* morphants displayed hypomorphic DA structures and no cardiac flow, the interrenal tissue in the *fn1* morphant was associated with the DA (Fig. 3B”, C”, E”, F”), but that in the *tnnt2a* morphant was attached to the IRV and away from the DA (Fig. 8A). It implicated a higher affinity of interrenal cells to the IRV than to the DA in the flow-deficient embryo, and that the blood flow might be required for interrenal cells to be attracted toward the sprouting site of IRV.

Accompanying with the altered IRV and interrenal phenotypes in the *tnnt2a* embryo, the peri-IRV Fn was diffusely distributed around the IRV, while Fn is tightly aggregated around the extending IRV in the control embryo (Fig. 8B). Fn is a key mechanosensitive ECM component and contains cryptic sites that require cell-generated mechanical force to unfold their structures [34], [35]. Such unfolding, particularly through integrin-based interactions, could promote self-assembly of Fn into fibrils. It remains unclear whether the Fn polymerization around the IRV was affected in the absence of blood flow, and whether it could modulate the direction of either IRV growth or interrenal morphogenetic movement. Nevertheless, the blood flow appeared to be essential for modulating the Fn-rich microenvironment, and an alteration of which might at least in part account for the aberrant IRV morphology and IRV-interrenal interaction in the *tnnt2a* morphant.

### The Administration of Fn Antagonist through Microangiography Reduced IRV Angiogenesis as well as Interrenal Cell Migration

To further demonstrate that the Fn signaling has a blood flow-independent effect on the IRV growth and the interrenal morphogenesis, we injected the Fn antagonist RGD heptapeptide, or the BSA as control, at the concentration of 10 mM (4.6 µl) into the circulation of *Tg(kdrl:GFP)^s843^* embryos at 1.5 dpf through microangiography. The injected embryos displayed no notable change in terms of either general growth or heart rate, and were collected for analysis at 52 hpf, a stage when the IRV has grown ventrally but not yet curved anteriorly; and so its length could be detected on the same focal plane (Fig. 8C). The DA structure showed no obvious defect in the RGD-treated embryo. However, the quantified length of the IRV in the RGD-treated embryo has been significantly reduced to 48% of that in the BSA-treated control (Fig. 8D). This indicated that the IRV phenotype in the *fn1* mutant or morphant could be directly attributed to a disruption of Fn signaling, but not secondary to early cardiovascular defects. An RGD injection at 32 hpf did not further enhance its effect on the IRV growth (data not shown). The efficiency of RGD peptides to target peri-IRV and/or peri-interrenal regions by microangiography is not determined yet, and thus awaits specific labeling and purification of RGD peptides; although indirect evidence showed that the rhodamine dextran co-injected with RGD peptides was detectable at the peri-IRV region (Fig. S5). On the other hand, the incomplete repression of IRV growth in the RGD-treated embryos suggested that the peptide competition to block the RGD-binding sites of Integrins could only partially inhibit the Fn signaling at the interrenal vascular microenvironment; which implicated a possibility that Fn might also interact with other proangiogenic factor(s) for promoting interrenal vascularization. Nevertheless, the results of both pharmaceutical RGD-peptide treatment and knockdown experiments targeting either *fn1* or *itga5* supported a role of Fn signaling for the IRV growth. Moreover, the migratory behavior of the steroidogenic interrenal tissue was affected in the RGD-administered embryo. While migrating tips of the interrenal tissue in the BSA-injected embryos have either reached or crossed the midline, those in the RGD-injected embryos did not reach the midline (Fig. 8C, E), suggesting a reduction in the migratory activity of interrenal cells in the RGD treatment. It was therefore concluded that Fn signaling might have direct regulatory role, independently of blood flow, on both the IRV growth and the interrenal migration. However, the blood flow might function to maintain the tight interaction among the growing IRV, the IRV-associated Fn and the interrenal cells, which was essential for dictating the correct direction of IRV growth, and the ability of interrenal cells to migrate toward and around the sprouting IRV.

## Discussion

While our earlier results show that morphogenetic movements of the interrenal tissue prior to the integration of steroidogenic and interrenal lineages are promoted by the peri-interrenal endothelium [5], [6], this study demonstrates the modulation of steroidogenic cell migration by the intrainterrenal vasculature and its associated ECM environment; at a stage when the interrenal gland is under active functional assembly. In this scenario, Fn plays a dual role by promoting both the IRV angiogenesis and the steroidogenic cell migration. The low percentage of interrenal midline fusion phenotype in the *fn1* mutant and morphant suggested that Fn could play a minor role in the endothelium-promoted morphogenetic movements prior to the interrenal organ assembly. In addition, the penetrance of interrenal midline fusion phenotype in embryos deficient in Fn, Itga5 or both is much lower than that in the endothelium-free *clo* mutant, suggesting that a disruption of Fn-Itga5 signaling might not fully represent the molecular mechanism underlying the early interrenal fusion phenotype in *clo*. Nevertheless, the *fn/itga5* double morphant demonstrated a higher penetrance of the interrenal midline fusion phenotype than that in either *fn* or *itga5* single morphant, indicating that prior to the functional organ assembly Fn might function redundantly in the early interrenal-endothelial interaction.

The spanning of IRV through the interrenal tissue occurred at around 2 dpf, and the intra-interrenal circulation was established by 3 dpf; which implicated an establishment of the hypothalamus-pituitary-interrenal axis only after 2 dpf. The temporal coincidence between the onset of intra-interrenal circulation and the integration between steroidogenic and chromaffin cell lineages reflects the maturation of a fully-functional interrenal gland system, for building up the stress axis in the embryo. This is consistent with the previous findings that zebrafish larvae are able to respond to an osmotic stressor as early as 3 dpf [36]. The architecture of IRV suggested a direct supply of steroids through the AMA and its derived vessels to the swim bladder and the gut, which implied a high demand of steroids during the development of these two organs. In the zebrafish, inflation of the swim bladder and maturation of the gastrointestinal system are two important characteristics featuring the transition from a yok-sac to a swimming larvae, which culminates in a switch from endogenous to exogenous nutrient uptake [37]. Indeed, severe deformities of both swim bladder and gut tube could be observed in the *ff1b* moprhant embryo where the interrenal tissue is fully ablated, as well as in the embryo treated with an inhibitor of steroid synthesis [38]. Hence, although species-specific differences exist between the fish interrenal and the mammalian adrenal glands in terms of their organ architectures and physiological functions, the remodeling of both organs during development are crucial for ensuring steroid homeostasis in early life.

In contrast to the rather simplistic pattern of interrenal vessel formation in the zebrafish, both adrenal cortex and medulla in mammals contain extensive vascular networks. Morphologically, the dense distribution of vasculature in the human adrenal cortex is derived from three sets of blood vessels for each adrenal gland, which include the inferior phrenic artery branching off the aorta, the middle suprarenal artery branching off the aorta, and the inferior suprarenal arteries branching off each renal artery [39], [2], [40]. How this well-organized vascular network is established during embryonic development had remained unclear. Albeit the evident difference between the mammalian adrenal gland and the teleostean interrenal gland development, the results in this study hinted that the interaction between the steroidogenic and endothelial cells during adrenal gland vascularization might require Fn and its interacting partner(s). Also, our histological and functional analyses suggested a tendency for steroidogenic cells to migrate toward the Fn-enriched vascular microenvironment, whose pattern could be modulated by the blood flow. This implies a possibility that parallel centripetal patterns of adrenocortical cell migration and vascularization in the fetal adrenal gland could be regulated by the Fn gradient where a rich deposition at the center of FZ is evident [41], and the blood flow might participate in patterning the Fn gradient as well as the vasculature architecture. It is interesting to note that in the adult mouse adrenal gland, Fn localizes restrictively around the vessel at the inner medulla region [42]. It however awaits future studies to determine whether the Fn gradient in the fetal adrenal cortex is derived from the permeating vessels. The ECM proteins Laminin, Collagen IV and Fn in the fetal adrenal gland demonstrate different modulating properties on the adrenal cells in the *in vitro* culture experiments [43], [4]. Laminin or Collagen IV-coated dishes enhance proliferation of primary fetal adrenal cells, while Laminin also protects these cells from apoptosis. Collagen IV also serves to enhance the cortisol secretion in response to the ACTH stimulus. Conversely, adrenal cells cultured on Fn show no proliferation, and fail to respond to ACTH for either steroidogenic gene expression or cortisol secretion. These primary culture data are consistent with our results in terms that Fn did not seem to play any role in promoting either cell proliferation or steroidogenic gene expression in the zebrafish interrenal tissue.

Laminin in the fetal adrenal gland displays a mirror-image distribution with that of Fn by being restrictively localized at the outer mitotically active DZ, and cooperates with Collagen IV to promote adrenocortical proliferation [43]. The Laminin-enriched DZ also shows a high angiogenic activity which leads to a network of capillaries at the periphery [44]. It remains unclear whether the Laminin and Collagen IV distribution at the periphery of fetal adrenal cortex is generated by the endothelium. So far, whether an equivalent region of DZ is present in the embryonic zebrafish interrenal gland remains unknown. However, it might be interesting to explore whether Laminin also plays a role in the fish interrenal morphogenesis. Previous genetic studies in the zebrafish reveal that Laminin α1 and 4 isoforms function redundantly for anigogenic ISV sprouts to migrate along the Laminin-rich intersegmental boundaries [45]. The zebrafish loci *grumpy* and *sleepy* encode *laminin β1* and *γ1*, and are important both for notochord differentiation and for ISV formation [46]. On the contrary, although Fn is also deposited along the intersegmental boundary, the loss of Fn1 did not lead to an inhibition of IRV growth (Fig. 2 and S2). We speculate that this might reflect a low demand of Fn1 in the ISV growth; or the function of *fn1* for ISV angiogenesis is redundant. It is also likely that in fish diverse composition of vascular microenvironment is involved in various aspects of organ angiogenesis, which could be exemplified by the fact that in zebrafish *collagen IV* has been identified to be expressed only at the adult retinal vasculature [47], thus might not be involved in either ISV or IRV angiogenesis.

Our results supported the notion that endothelium-derived Fn cooperates with its putative receptor Itga5 to promote both migration and angiogenesis of the interrenal tissue. However, it is noteworthy that *fn* and *itga5* might be expressed by endothelial and peri-vascular cells respectively, and so it remains to be determined how Fn is involved in the proangiogenic signal transduction pathway that mediates the IRV growth. Previous work on the *in vitro* breast cancer cell line model shows that the function of VEGF_165_ in terms of mitogenic and migratory effects requires the presence of Fn and heparins [48]. Fn could physically interact with VEGF, and the association between VEGF and Fn is required for the full effects of VEGF-mediated endothelial cell migration and proliferation [49], [50]. Vascularization of the adrenal tissue requires the existence of proangiogenic molecules such as EG-VEGF (endocrine-gland-derived VEGF; or prokineticin 1) [51], Angiopoietin2 [52], [44], and VEGF [53]. EG-VEGF was identified for its tissue-specific angiogenic property to stimulate proliferation and migration in ECs isolated from steroidogenic organs including testis, ovary and adrenal gland. Although it remains unknown whether EG-VEGF would physically interact with Fn, EG-VEGF binds to heparin *in vitro*, and is expected to be sequestered in the extracellular compartment [51]. Angiopoietin2 and VEGF are expressed and secreted by the fetal adrenal cortex and are locally up-regulated by adrenocorticotropin which also controls adrenal organ growth [54], [52], indicating a dual hormonal regulation of adrenal tissue mass and vasculature. The pituitary control of interrenal cell growth does not initiate in the zebrafish embryo until 5 dpf [9], implying that the expression of proangiogenic factor(s) that induce the IRV formation might not be under the hormonal regulation; instead, they could be autonomously induced within the developing interrenal tissue. It is possible that such proangiogenic factor(s) would enable the embryonic interrenal tissue to attract ECs and induce the sprouting of IRV and the concomitant synthesis of Fn. The Fn deposition around the IRV in turn provides an environment to support the further growth of IRV, plausibly through its physical interaction with proangiogenic factors. Meanwhile, the peri-IRV Fn accumulation also interacts with the Itga5 molecules expressed by the surrounding interrenal cells, thus activating the Integrin-mediated pathway to mediate interrenal morphogenesis. In this scenario, the Fn microenvironment plays a dual role in both IRV growth and interrenal morphogenesis. In the future, the identification and characterization of proangiogenic factor(s) that induce the IRV growth will help to clarify whether and how they induce and interact with the Fn molecules.

Identification of the zebrafish IRV in our study also enhances the capacity of utilizing the zebrafish embryo as an *in vivo* platform for studying organ-specific angiogenesis. Our study shows that the Fn antagonist treatment through microangiography could effectively reduce the angiogenic growth and interrenal migration, indicating that the zebrafish interrenal gland could be a potential system for testing and quantifying the efficacy of peptide drugs in terms of the anti-angiogenesis properties.

## Supporting Information

Figure S1 **The IRV is sprouted from the DA and connected to the AMA.** (A) Sets of confocal images display the interrenal tissues (IR, white arrows) as detected by 3β-Hsd activity staining, and the neighboring endothelium as labeled by green fluorescence, of *Tg(kdrl:EGFP)^s843^* embryos at 45, 69, and 84 hpf respectively. Panels (a, a’) are dorsolateral while panels (b, b’, c, c’) are lateral views, and all panels are oriented with anterior to the right. The fluorescent image of the vascular pattern for the 45 hpf embryo (a’) was acquired through a projection of a consecutive z-stack encompassing the peri-interrenal area, while single confocal images were shown for the peri-interrenal vascular patterns at 69 and 84 hpf (b’, c’). The IRV was formed caudal to and distinct from the pronephric glomerulus (G) and the AMA. Red and blue arrows denote arterial and venous structures, respectively. (B) The transverse view of the vascular structure neighboring the IRV. The fluorescent image represents a projection of a consecutive z-stack encompassing the IRV, and the more posterior swim bladder artery (SBA) segments. The IRV sprouted from the ventral DA and connected to the AMA segment near which two branches of SBA were branched out. The rotation view of this projection is shown in Video S1. (C) Microangiography by injecting rhodamine-dextran into the blood stream of a *Tg(ff1bExon2:GFP)* embryo at 3 dpf. The blood circulation through the developing interrenal tissue is established by 3 dpf. Abbreviations: interrenal tissue (IR), dorsal aorta (DA), intersegmental vessel (ISV), interrenal vessel (IRV), glomerulus (G), posterior cardinal vein (PCV), common cardinal vein (CCV), anterior mesenteric artery (AMA), SBA (swim bladder artery).
(TIF)

Figure S2 **The phenotype of ISV and SIV in the** ***fn1*** **morphant.** Confocal images display lateral views of the ISV (5^th^ and 6^th^ pairs denoted by red arrows) and SIV (bracketed by red lines) of 3 dpf *Tg(kdrl:EGFP)^s843^* embryos injected with either STD-MO (upper panel) or *fn1*MO (lower panel), and the anterior is oriented to the right. The first four pairs of ISV (indicated by red arrowheads) and the SIV display aberrant angiogenic patterns in the absence of Fn. Abbreviations: intersegmental vessel (ISV), subintestinal vessel (SIV).
(TIF)

Figure S3 **The expression of** ***fn1b*** **could not be detected in the vascular region during the growth of IRV.** Unlike *fn1* transcripts (black; indicated by white arrowhead in the upper panel) which could be detected around and ventral to the DA on the transverse section at the level of *ff1b*-expressing interrenal tissue (red) in a 2 dpf embryo, no *fn1b* mRNA is present at the same area (lower panel). Abbreviations: notochord (NC), dorsal aorta (DA), swim bladder (SB).
(TIF)

Figure S4 **The distribution of pFAK in the interrenal and peri-interrenal regions of the** ***fn1*** **and** ***itga5*** **morphants as well as the wild-type control embryo at 2.5 dpf.** Transverse sections of *fn1* morphants (B, B’, C, C’) and *itga5* morphants (D, D’, E, E’), as well as the wild type control embryo (A, A’), which were assayed for 3βHsd activity (black) and pFAK expression (red). All sections are oriented with the posterior end toward top of page. While pFAK could be readily detected in both interrenal and peri-interrenal regions of the wild-type embryo, its presence was disrupted in either *fn1* or *itga5* morphants. The 3βHsd-expressing interrenal tissues are indicated by organge arrows. Abbreviations: notochord (NC), swim bladder (SB).
(TIF)

Figure S5 **The distribution of rhodamine dextran as coinjected with RGD peptides into the circulation.** The rhodamine dextran (red) could be detected around the IRV in the *Tg(kdrl:EGFP)^s843^* embryo as harvested at 52 hpf (indicated by yellow arrowheads), after being co-injected with RGD peptides by microangiography at 1.5 dpf. Abbreviations: notochord (NC), interrenal tissue (IR), interrenal vessel (IRV).
(TIF)

Video S1 Three-dimensional rotation view from a Z-stack projection of the confocal stack used to generate Fig. S1B. The z-stack consists of 64 optical sections (z-increments of 3 µM) at a pixel density of 1024×1024.
(MPG)
