# Supplementary figures and images for: Development and Fibronectin Signaling Requirements of the Zebrafish Interrenal Vessel
Source: PLoS One. 2012 Aug 21;7(8):e43040. doi: 10.1371/journal.pone.0043040 (PMC3428036; doi:10.1371/journal.pone.0043040)

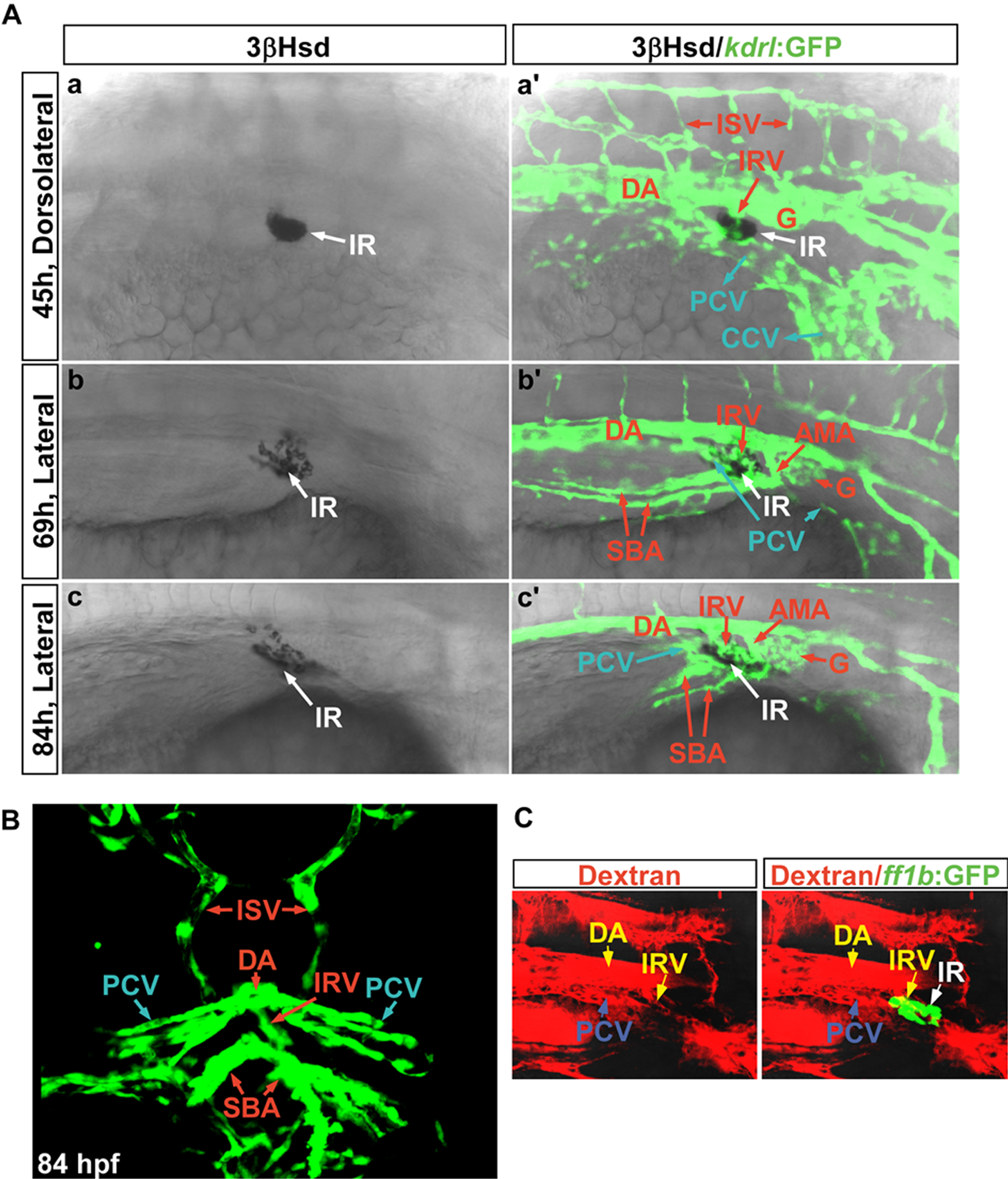

Supplement: Figure S1 — The IRV is sprouted from the DA and connected to the AMA. (A) Sets of confocal images display the interrenal tissues (IR, white arrows) as detected by 3β-Hsd activity staining, and the neighboring endothelium as labeled by green fluorescence, of Tg(kdrl:EGFP)s843 embryos at 45, 69, and 84 hpf respectively. Panels (a, a’) are dorsolateral while panels (b, b’, c, c’) are lateral views, and all panels are oriented with anterior to the right. The fluorescent image of the vascular pattern for the 45 hpf embryo (a’) was acquired through a projection of a consecutive z-stack encompassing the peri-interrenal area, while single confocal images were shown for the peri-interrenal vascular patterns at 69 and 84 hpf (b’, c’). The IRV was formed caudal to and distinct from the pronephric glomerulus (G) and the AMA. Red and blue arrows denote arterial and venous structures, respectively. (B) The transverse view of the vascular structure neighboring the IRV. The fluorescent image represents a projection of a consecutive z-stack encompassing the IRV, and the more posterior swim bladder artery (SBA) segments. The IRV sprouted from the ventral DA and connected to the AMA segment near which two branches of SBA were branched out. The rotation view of this projection is shown in Video S1. (C) Microangiography by injecting rhodamine-dextran into the blood stream of a Tg(ff1bExon2:GFP) embryo at 3 dpf. The blood circulation through the developing interrenal tissue is established by 3 dpf. Abbreviations: interrenal tissue (IR), dorsal aorta (DA), intersegmental vessel (ISV), interrenal vessel (IRV), glomerulus (G), posterior cardinal vein (PCV), common cardinal vein (CCV), anterior mesenteric artery (AMA), SBA (swim bladder artery). (TIF) [file pone.0043040.s001.tif]

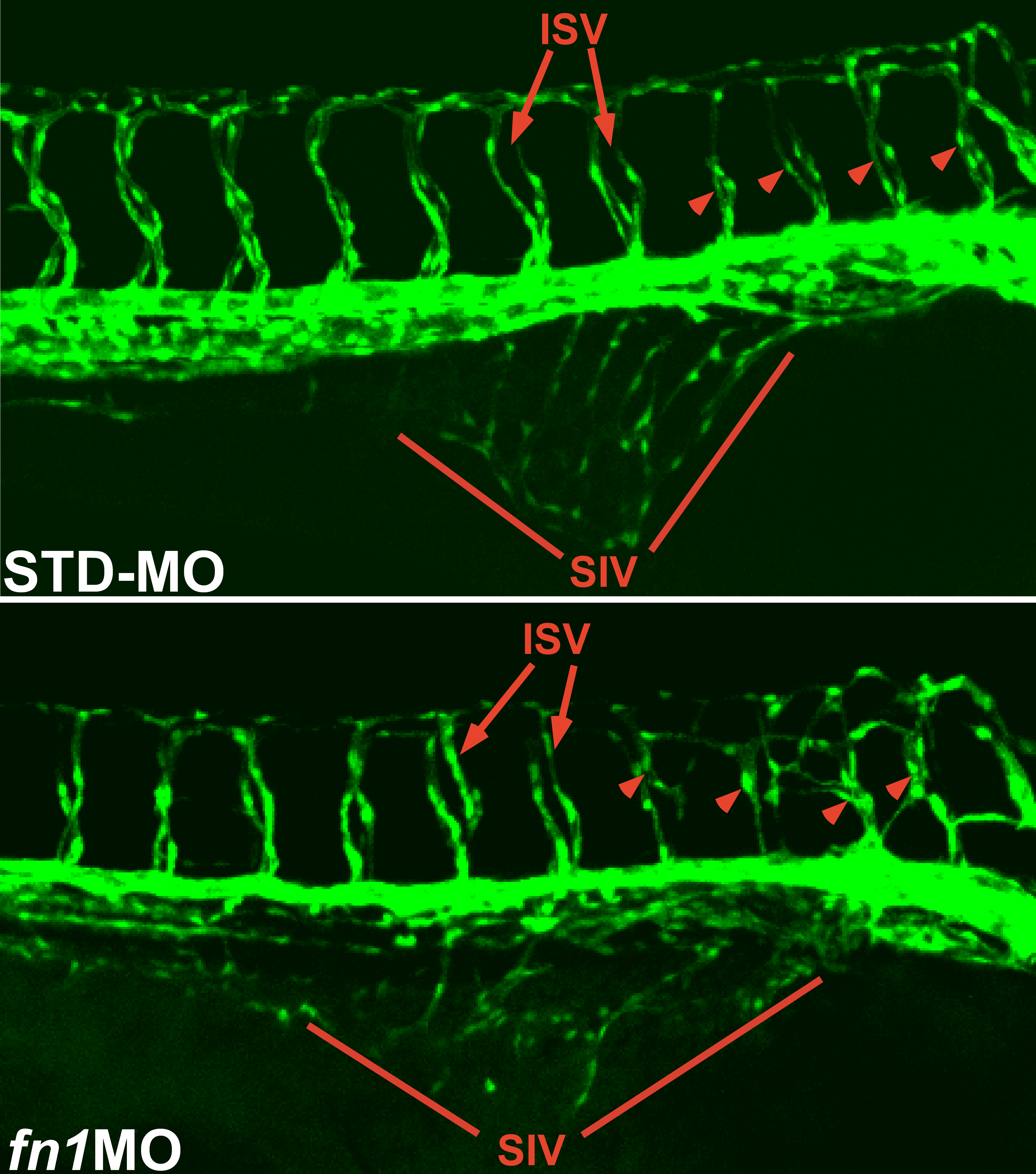

Supplement: Figure S2 — The phenotype of ISV and SIV in the fn1 morphant. Confocal images display lateral views of the ISV (5th and 6th pairs denoted by red arrows) and SIV (bracketed by red lines) of 3 dpf Tg(kdrl:EGFP)s843 embryos injected with either STD-MO (upper panel) or fn1MO (lower panel), and the anterior is oriented to the right. The first four pairs of ISV (indicated by red arrowheads) and the SIV display aberrant angiogenic patterns in the absence of Fn. Abbreviations: intersegmental vessel (ISV), subintestinal vessel (SIV). (TIF) [file pone.0043040.s002.tif]

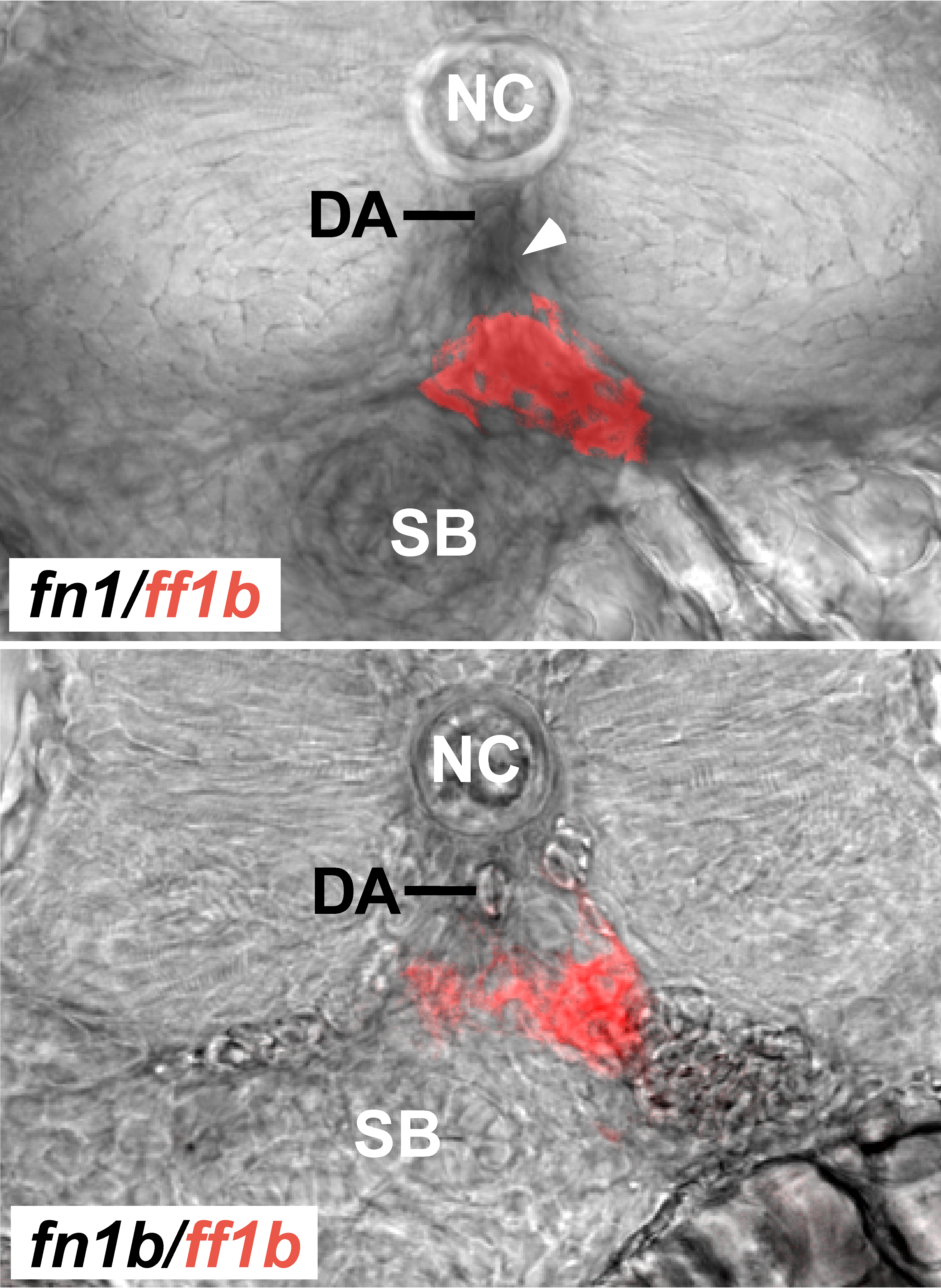

Supplement: Figure S3 — The expression of fn1b could not be detected in the vascular region during the growth of IRV. Unlike fn1 transcripts (black; indicated by white arrowhead in the upper panel) which could be detected around and ventral to the DA on the transverse section at the level of ff1b-expressing interrenal tissue (red) in a 2 dpf embryo, no fn1b mRNA is present at the same area (lower panel). Abbreviations: notochord (NC), dorsal aorta (DA), swim bladder (SB). (TIF) [file pone.0043040.s003.tif]

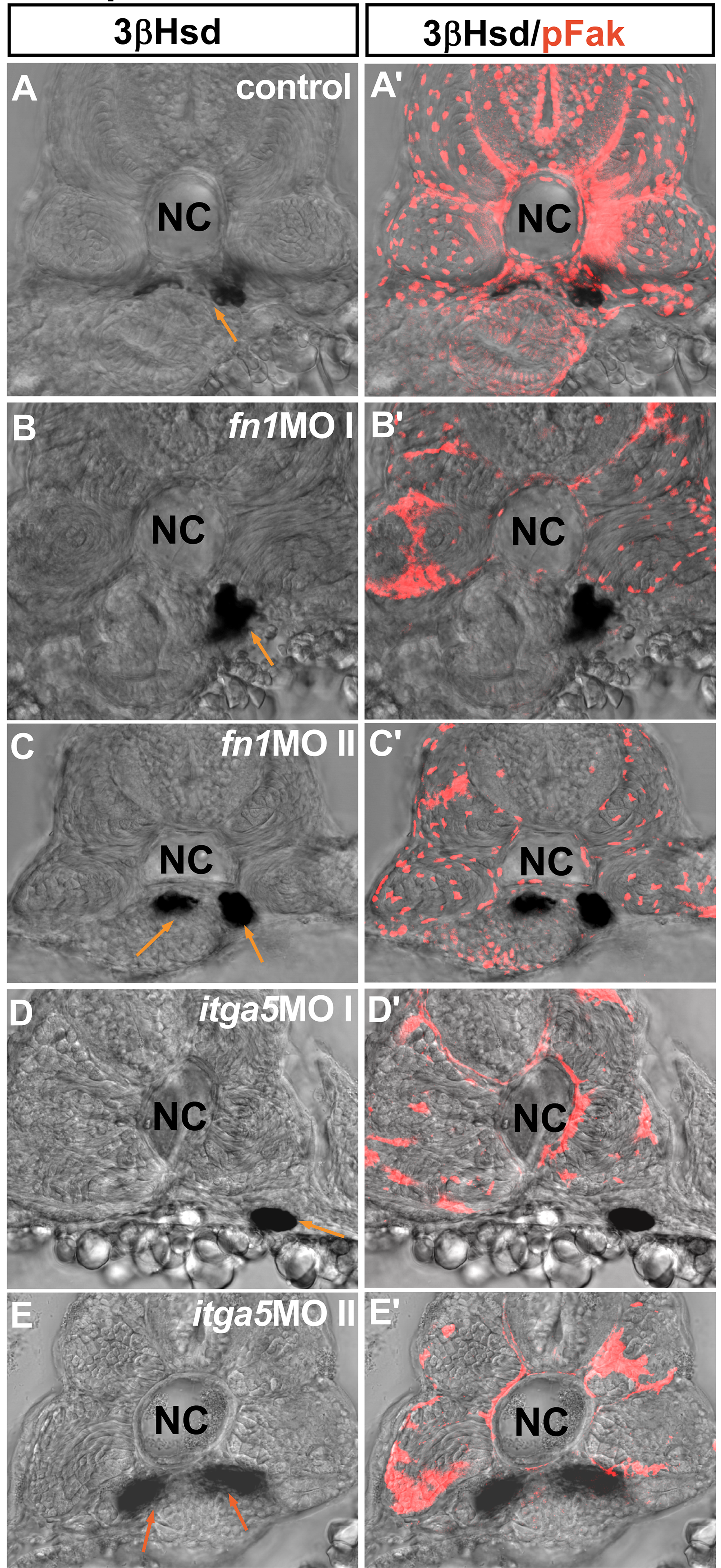

Supplement: Figure S4 — The distribution of pFAK in the interrenal and peri-interrenal regions of the fn1 and itga5 morphants as well as the wild-type control embryo at 2.5 dpf. Transverse sections of fn1 morphants (B, B’, C, C’) and itga5 morphants (D, D’, E, E’), as well as the wild type control embryo (A, A’), which were assayed for 3βHsd activity (black) and pFAK expression (red). All sections are oriented with the posterior end toward top of page. While pFAK could be readily detected in both interrenal and peri-interrenal regions of the wild-type embryo, its presence was disrupted in either fn1 or itga5 morphants. The 3βHsd-expressing interrenal tissues are indicated by organge arrows. Abbreviations: notochord (NC), swim bladder (SB). (TIF) [file pone.0043040.s004.tif]

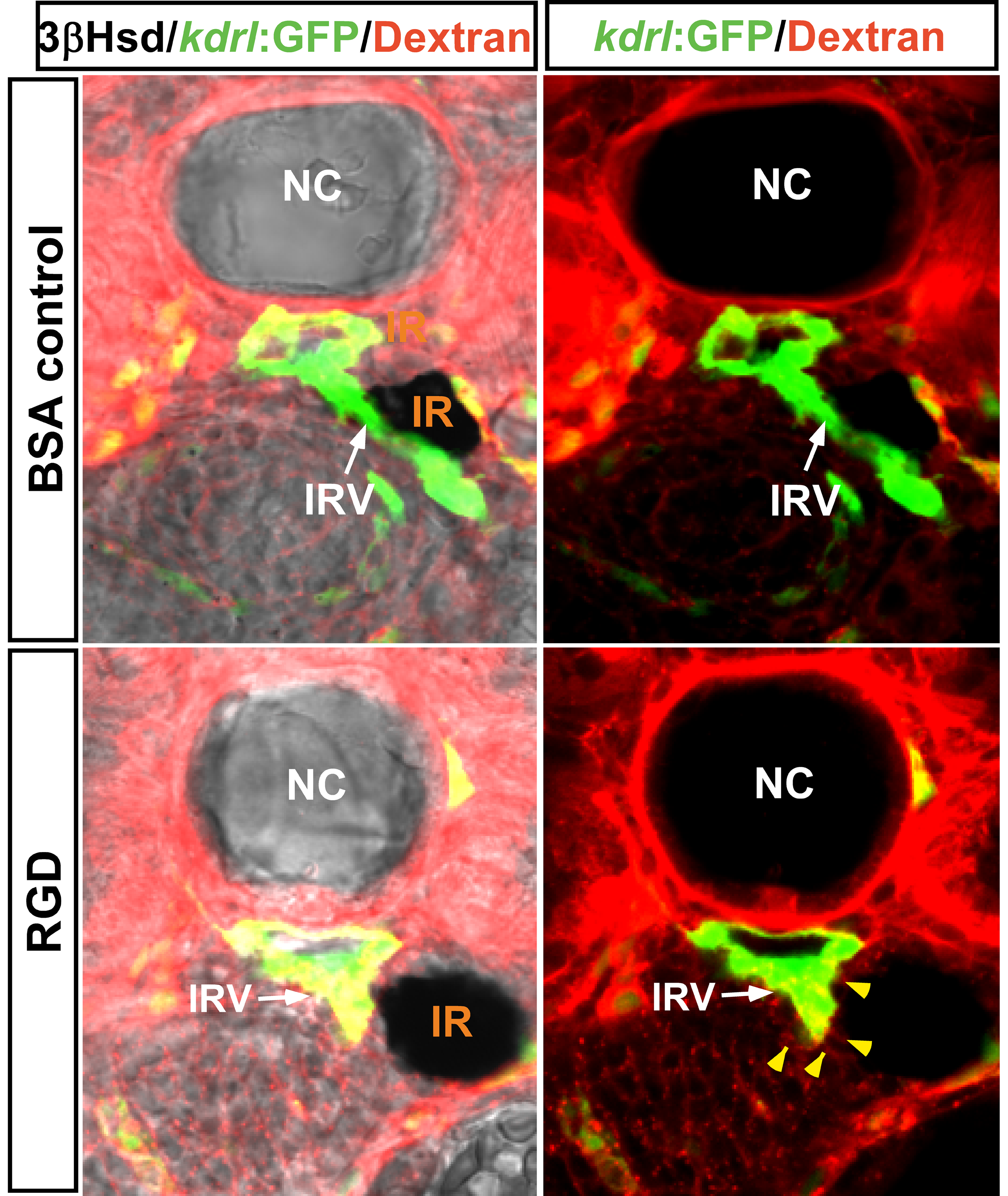

Supplement: Figure S5 — The distribution of rhodamine dextran as coinjected with RGD peptides into the circulation. The rhodamine dextran (red) could be detected around the IRV in the Tg(kdrl:EGFP)s843 embryo as harvested at 52 hpf (indicated by yellow arrowheads), after being co-injected with RGD peptides by microangiography at 1.5 dpf. Abbreviations: notochord (NC), interrenal tissue (IR), interrenal vessel (IRV). (TIF) [file pone.0043040.s005.tif]
